# In silico approach to the analysis of SNPs in the human APAF1 gene

**DOI:** 10.3906/biy-1905-18

**Published:** 2019-12-13

**Authors:** Tuğba KAMAN, Ömer Faruk KARASAKAL, Ebru ÖZKAN OKTAY, Korkut ULUCAN, Muhsin KONUK

**Affiliations:** 1 Department of Medicinal and Aromatic Plants, Vocational School of Health Services, Üsküdar University, İstanbul Turkey; 2 Department of Medical Laboratory Techniques, Vocational School of Health Services, Üsküdar University, İstanbul Turkey; 3 Department of Laboratory Technology, Üsküdar University, Vocational School of Health Services, Üsküdar, İstanbul Turkey; 4 Department of Molecular Biology and Genetics, Faculty of Engineering and Natural Sciences, Üsküdar University, İstanbul Turkey; 5 Department of Medical Biology and Genetics, Faculty of Dentistry, Basic Medical Sciences, Marmara University, İstanbul Turkey

**Keywords:** *APAF1*, apoptosis, neurodegenerative diseases, single nucleotide polymorphism (SNP), in silico

## Abstract

The apoptotic protease activating factor 1 (*APAF1*) gene encodes a cytoplasmic protein that initiates apoptosis and is a crucial factor in the mitochondria-dependent death pathway. *APAF1* is implicated in many pathways such as apoptosis, neurodegenerative diseases, and cancer. The purpose of this study was to predict deleterious/damaging SNPs in the *APAF1* gene via**in silico**analysis. To this end, *APAF1* missense SNPs were obtained from the NCBI dbSNP database*. *In silico analysis of the missense SNPs was carried out by using publicly available online software tools. The stabilization and three-dimensional modeling of mutant proteins were also determined by using the I-Mutant 2.0 and Project HOPE webservers, respectively. In total, 772 missense SNPs were found in the *APAF1* gene from the NCBI dbSNP database, 18 SNPs of which were demonstrated to be deleterious or damaging. Of those, 13 SNPs had a decreasing effect on protein stability, while the other 5 SNPs had an increasing effect. Based on the modeling results, some dissimilarities of mutant type amino acids from wild-type amino acids such as size, charge, and hydrophobicity were revealed. The SNPs predicted to be deleterious in this study might be used in the selection of target SNPs for genotyping in disease association studies. Therefore, we could suggest that the present study could pave the way for future experimental studies.

## 1. Introduction

Apoptosis is the programmed cell death process that is required for tissue homeostasis and development. It elicits a cellular response to stress and pathogens as well. The disruption of the apoptosis process can lead to human diseases such as cancer, neurodegenerative disorders, heart disease, autoimmunity, and immunodeficiencies (u1d6a). After Bax activation, cytochrome c is released from the mitochondrial intermembrane space. Cytochrome c and ATP/dATP then interact with APAF1, resulting in the formation of apoptosome, which binds and activates procaspase-9, which in turn activates caspase 3, thereby leading cells into apoptosis (Li et al., 1997; u1d6d; u1d6e). APAF1 plays a key role in the intrinsic or mitochondrial pathway of apoptosis. Therefore, APAF1 is related to regulation and function in cell death (Shakeri et al., 2017). The *APAF1* gene is implicated in various pathophysiological pathways such as platinum drug resistance, apoptosis, and neurodegenerative diseases—e.g., Alzheimer’s disease, amyotrophic lateral sclerosis (ALS), Parkinson’s disease, Huntington’s disease, legionellosis, tuberculosis, hepatitis B, and cancer (https://www.genome.jp/).

APAF1 protein contains 3 functional regions that are involved in protein–protein interactions. The caspase recruitment domain (CARD) is located at the N-terminus of the protein, and it is very important for the interaction of APAF1 with caspase-9 (Riedl and Salvesen, 2007). CARD is followed by a nucleotide-binding and oligomerization domain (NOD, also known as NB-ARC). NB-ARC is an ATPase domain that causes a conformational change in APAF1 during apoptosis (Bratton and Salvesen, 2010). WD repeats domains, located at the C-terminus of the protein, are responsible for cytochrome c binding (Reed et al., 2004; Riedl and Salvesen, 2007).

There are various functional classes of single nucleotide polymorphisms (SNPs) such as 3’ splice site, 3’ UTR (untranslated region), 5’ splice site, 5’ UTR, coding synonymous, frameshift, intron, missense, nonsense, and gained. Among these, missense SNPs cause amino acid substitutions. The effect of amino acid substitutions on protein structure and function is critical for understanding the complex mechanisms of human diseases caused by single amino acid mutations (Cargill et al., 1999; Teng et al., 2010). SNPs are known biomarkers for predicting disease susceptibility. Studies on SNPs in candidate genes associated with diseases have witnessed a remarkable increase in recent years. The association of SNPs in the *APAF1* gene with diseases has also been investigated recently (Ester et al., 2007; Enjuanes et al., 2008; u1d71; u1d72; u1d73). According to published research studies, the damaging effects of missense SNPs in the *APAF1* gene have not yet been studied using in silico methods. 

In this study, we aimed to detect missense SNPs in the *APAF1* gene to investigate the possible effects of SNPs on the physicochemical properties of the amino acid residues of APAF1 (such as size, charge, hydrophobicity, structure, domain, and conservation), as well as the APAF1 protein structure, by using bioinformatics methods.

## 2. Materials and methods

### 2.1. Data mining

The NCBI dbSNP database was used to access the SNPs in the *APAF1* gene (NCBI Gene ID: 317) in July 2018 https://www.ncbi.nlm.nih.gov/snp. Missense SNPs cause changes in the amino acid sequence of the corresponding gene and may have an impact on the function of the given protein. For this reason, the missense SNPs in the *APAF1 *gene were selected for analysis. The primary sequence of the protein (Uniprot accession number: O14727) encoded by the *APAF1* gene was obtained from the UniProt database u1fdb.

### 2.2 Bioinformatics data analysis

**Figure 1:**
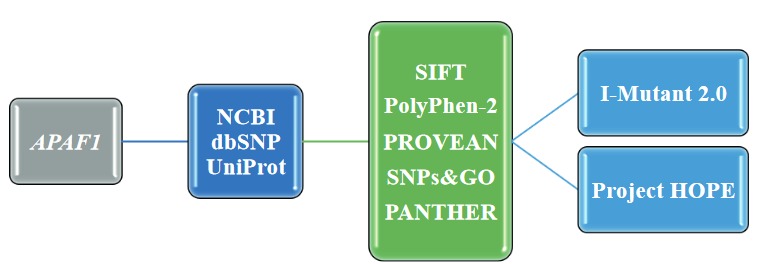
Usage of online software tools in SNP analyses (modified from Marwa Mohamed Osman et al., 2016).

Online software tools were used to determine the effects of *APAF1* SNPs on the APAF1 protein through the use of three-dimensional models of mutant proteins (Figure 1) (u1d74; u1d75; u1d76; u1d77; u1d78). The effects of the missense SNPs in the *APAF1* gene were assessed using SIFT (Sorting Intolerant from Tolerant), PolyPhen-2 (Polymorphism Phenotyping v2), SNPs & GO, PROVEAN (Protein Variation Effect Analyzer), and PANTHER online software tools. SIFT estimates the effects of an amino acid replacement on the function of a protein based on the sequence similarity and physical features of amino acids u1fdd. PolyPhen-2 is a software tool that predicts the effects of an amino acid replacement on the structure and function of a given protein based on physical and comparative properties u1fdf. SNPs & GO predicts whether a single nucleotide polymorphism is disease-related using protein functional annotation u1fe1. PROVEAN is a software tool that estimates the possible effect of an amino acid replacement on the protein function u1fe3. PANTHER estimates the potential effect of a missense SNP on the function of a given protein u1fe5. The SNPs predicted to be deleterious by all of the software tools used were selected for further analysis. The effect of these SNPs on the protein stabilization was determined via I-Mutant 2.0 u1fe7. Three-dimensional modeling of mutant proteins was performed with the Project HOPE software tool u1fe9

### 2.3 Gene–gene interactions

Additionally, gene–gene networks including known and predicted gene interactions from different sources such as gene coexpression, colocalization, and shared protein domains were investigated by using the GeneMANIA software tool u1fec. 

## 3. Results

### 3.1. Gene–gene interactions

**Figure 2:**
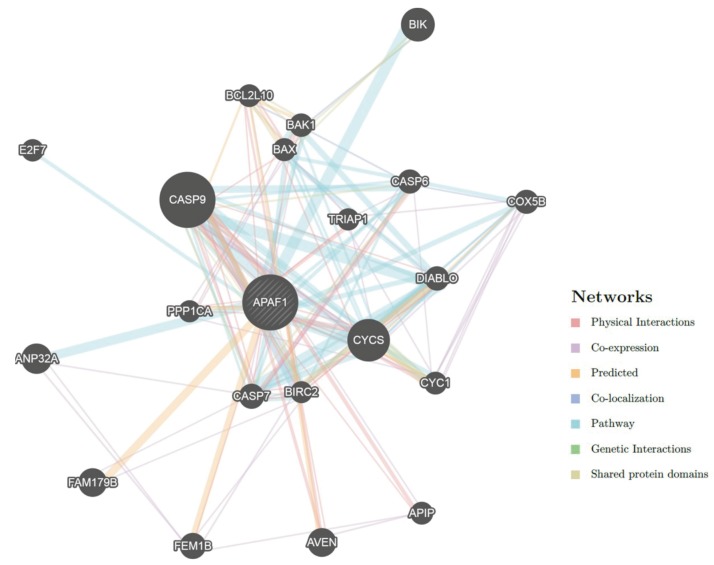
Gene–gene interactions of APAF1.

The gene–gene interaction network of the *APAF1* gene is shown in Figure 2. The *APAF1* gene is linked to 20 genes in a highly interconnected network, with 170 edges10. 

### 3.2. Prediction of the effect of APAF1 SNPs on APAF1 protein structure

**Figure 3:**
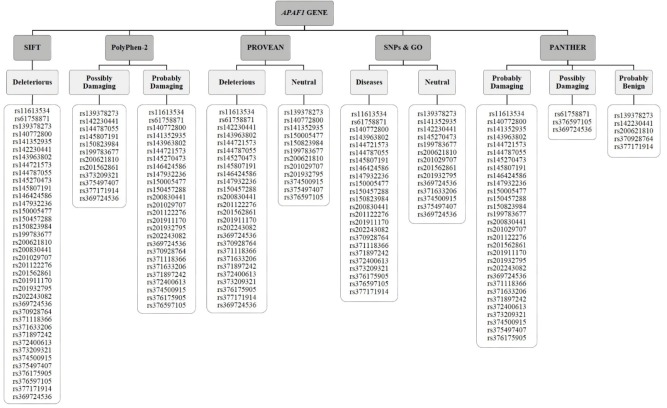
Deleterious or damaging SNPs predicted by online software tools.

According to the data obtained from the NCBI dbSNP database, there were a total of 18084 SNPs in the *APAF1* gene. Of those, 772 SNPs were found to be missense. First, 772 missense SNPs were submitted to the SIFT software tool; 38 SNPs were found to be deleterious (Figure 3). These 38 SNPs were then submitted to the PolyPhen-2 software tool; 12 SNPs were detected as possibly damaging, while the other 26 SNPs were found to be probably damaging (Figure 3). The 38 SNPs determined by the SIFT software tool to be deleterious were uploaded to the PROVEAN, SNPs & GO, and PANTHER software tools, respectively. We obtained 26 SNPs that were determined by PROVEAN to have no effect on protein stability (Figure 3). According to the SNPs & GO results, 24 SNPs were found to be associated with different diseases, although the other 14 SNPs were not predicted to have any effect (Figure 3). Moreover, 30 SNPs were predicted as probably damaging, 3 SNPs were predicted as possibly damaging, and 5 SNPs were found to be probably benign via the PANTHER software tool (Figure 3). Finally, 18 SNPs were predicted to be deleterious/damaging or disease-related via all of the online software tools (Table 1). In addition, the I-Mutant 2.0 software tool was used to evaluate the effects of damaging/deleterious SNPs on protein stabilization. The results showed that 13 SNPs have a decreasing effect on the stability of APAF1, while the other 5 SNPs have an increasing effect (Table 1).

**Table 1:** Results of SIFT, POLYPHEN-2, PROVEAN, SNPs & GO, PANTHER, and I- MUTANT software tools.

SNP ID	Nucleotide Change	Amino Acid Change	SIFT Result	SIFT Score	POLYPHEN-2 Result	POLYPHEN-2 Score	PROVEAN Result	PROVEAN Score	SNPs & GO	PANTHER Result	I-Mutant Result	I-Mutant RI
rs11613534	A/C (FWD)	A580D	Deleterious	0	Probably damaging	1.000	Deleterious	–4.46	Disease	Probably damaging	Decrease	6
rs61758871	A/C (FWD)	Y619S	Deleterious	0.025	Probably damaging	0.997	Deleterious	–3.57	Disease	Possibly damaging	Decrease	6
rs143963802	C/T (FWD)	A494V	Deleterious	0	Probably damaging	1.000	Deleterious	–3.75	Disease	Probably damaging	Increase	2
rs144721573	A/G (FWD)	D443N	Deleterious	0	Probably damaging	1.000	Deleterious	–3.17	Disease	Probably damaging	Decrease	6
rs144787055	G/T (FWD)	D170E	Deleterious	0.013	Possibly damaging	0.892	Deleterious	–2.61	Disease	Probably damaging	Increase	5
rs145807191	C/T (FWD)	S860L	Deleterious	0.004	Possibly damaging	0.954	Deleterious	–3.49	Disease	Probably damaging	Decrease	3
rs146424586	A/G (FWD)	H521R	Deleterious	0.024	Probably damaging	1.000	Deleterious	–3.93	Disease	Probably damaging	Decrease	4
rs147932236	C/T (FWD)	S161F	Deleterious	0.02	Probably damaging	0.999	Deleterious	–4.54	Disease	Probably damaging	Increase	3
rs150457288	A/G (FWD)	Y24C	Deleterious	0	Probably damaging	1.000	Deleterious	–4.42	Disease	Probably damaging	Increase	3
rs200830441	A/G (FWD)	H183R	Deleterious	0.009	Probably damaging	0.999	Deleterious	–3.83	Disease	Probably damaging	Decrease	3
rs201122276	A/G (FWD)	H1002R	Deleterious	0.024	Probably damaging	0.997	Deleterious	–5.58	Disease	Probably damaging	Decrease	4
rs201911170	A/G (FWD)	G951E	Deleterious	0.005	Probably damaging	0.999	Deleterious	–5.62	Disease	Probably damaging	Decrease	0
rs202243082	A/C (FWD)	P559T	Deleterious	0.011	Probably damaging	1000	Deleterious	–5.44	Disease	Probably damaging	Decrease	8
rs371118366	C/T (FWD)	C401R	Deleterious	0.027	Probably damaging	1000	Deleterious	–2.69	Disease	Probably damaging	Increase	0
rs371897242	C/T (FWD)	R612C	Deleterious	0.001	Probably damaging	1000	Deleterious	–4.79	Disease	Probably damaging	Decrease	5
rs372400613	A/T (FWD)	F544I	Deleterious	0.007	Probably damaging	1.000	Deleterious	–4.08	Disease	Probably damaging	Decrease	7
rs373209321	A/G (FWD)	D479G	Deleterious	0	Possibly damaging	0.932	Deleterious	–2.57	Disease	Probably damaging	Decrease	5
rs376175905	A/T (FWD)	F547I	Deleterious	0	Probably damaging	1000	Deleterious	–4.35	Disease	Probably damaging	Decrease	6

PolyPhen-2 score ≥0.5 = probably/possibly damaging; SIFT score ≤0.05 = deleterious , >0.05 = tolerated.

### 3.3. Modeling of amino acid substitutions caused by SNPs

Amino acids have physicochemical properties such as hydrophobicity value, charge, and size. Project HOPE showed that some of these features differed between wild-type and mutant-type amino acids, as shown in Table 2. In addition, 3-D APAF1 protein models generated based on the presence of *APAF1* SNPs are given in Table 3. Further information obtained from the Project HOPE software on the structure, domain, and conservation of 18 deleterious SNPs is given in Table 4.

**Table 2:** Results of wild-type and mutant-type amino acid properties obtained from Project Hope software.

SNP ID	AMINO ACID CHANGE	WILD-TYPE AMINO ACIDS			MUTANT TYPE AMINO ACIDS		
		Size	Charge	Hydrophobicity	Size	Charge	Hydrophobicity
rs11613534	A580D	<	neutral	>	>	– charge	<
rs61758871	Y619S	>			<		
rs143963802	A494V	<			>		
rs144721573	D443N		– charge			neutral	
rs144787055	D170E	<			>		
rs145807191	S860L	<		<	>		>
rs146424586	H521R	<	neutral		>	+ charge	
rs147932236	S161F	<		<	>		>
rs150457288	Y24C	>		<	<		>
rs200830441	H183R	<	neutral		>	+ charge	
rs201122276	H1002R	<	neutral		>	+ charge	
rs201911170	G951E	<	neutral	>	>	– charge	<
rs202243082	P559T			>			<
rs371118366	C401R	<	neutral	>	>	+ charge	<
rs371897242	R612C	>	+ charge	<	<	neutral	>
rs372400613	F544I	>			<		
rs373209321	D479G	>	– charge	<	<	neutral	>
rs376175905	F547I	>			<		

**Table 4:** Results of the 3D models of the APAF1 protein via Project Hope.

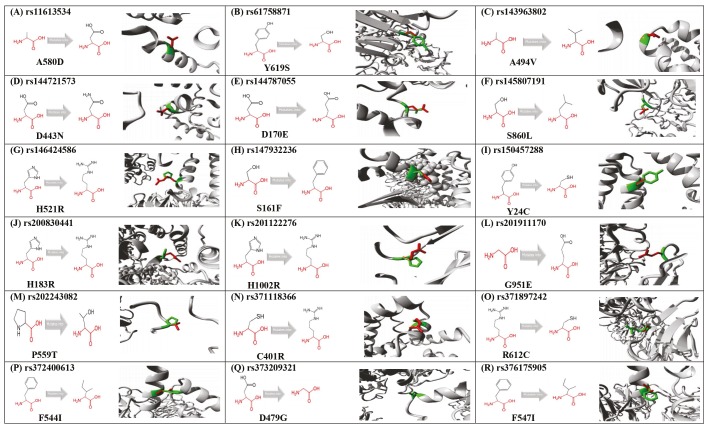

**Table 5:** Effects of amino acid changes on APAF1 protein from Project Hope.

SNP ID	Amino acid change	Structure	Domain	Conservation
rs11613534	A580D	Wild-type amino acid is found in an α-helix opposite the mutant amino acid.	Wild-type amino acid is embedded in the core of a domain, and the mutation can disrupt its structure.	The amino acid is 100% conserved in this location. Thus, the mutation is generally harmful to the protein.
rs61758871	Y619S	The mutant residue is found in WD 1-1 repeated residues.	The domain in which the amino acid at this position is located has an effect on binding to other molecules and interacts with a domain that is also involved in binding. The mutation of this position can disrupt these interactions and the function ofthe protein.	The wild-type amino acid is very conserved; however, another amino acid type may be found at this location. Thus, the mutation is likely not harmful to the protein.
rs143963802	A494V	Wild-type amino acid is found in an α-helix opposite to mutant amino acid.	The amino acid is embedded in the core of a domain which can be disrupted due to mutation at this position.	The amino acid is 100% conserved in this location. Thus, the mutation is generally harmful to the protein.
rs144721573	D443N		The amino acid is embedded in the core of a domain which can be disrupted due to mutation at this position.	The amino acid is 100% conserved in this location. Thus, the mutation is generally harmful to the protein.
rs144787055	D170E	The mutation occurs within the NB-ARC (UniProt) domain.	The domain in which the amino acid at this position is located has an effect on binding to other molecules and interacts with a domain that is also involved in binding. The mutation of this position can disrupt these interactions and the function ofthe protein.	The wild-type amino acid is very conserved; however, another amino acid type may be found at this location. Thus, the mutation is likely not harmful to the protein.
rs145807191	S860L	The mutant residue is found in WD 1-6 repeated residues.	The domain in which the amino acid at this position is located has an effect on binding to other molecules and interacts with a domain that is also involved in binding. The mutation of this position can disrupt these interactions and the function ofthe protein.	The amino acid is 100% conserved in this location. Thus, the mutation is generally harmful to the protein.
rs146424586	H521R		The amino acid at this position is situated on the surface of a domain that has unknown function.	The wild-type amino acid is very conserved; however, another amino acid type may be found at this location. Thus, the mutation is likely not harmful to the protein.
rs147932236	S161F	The mutation occurs within the NB-ARC (UniProt) domain.	The domain in which the amino acid at this position is located has an effect on binding to other molecules and interacts with a domain that is also involved in binding. The mutation of this position can disrupt these interactions and the function ofthe protein.	The wild-type amino acid is very conserved; however, another amino acid type may be found at this location. Thus, the mutation is likely not harmful to the protein.
rs150457288	Y24C	The mutation occurs within the CARD (UniProt) domain.	The amino acid is embedded in the core of a domain which can be disrupted due to mutation at this position.	The wild-type amino acid is very conserved; however, another amino acid type may be found at this location. Thus, the mutation is likely not harmful to the protein.
rs200830441	H183R	The mutation occurs within the NB-ARC (UniProt) domain.	The domain in which the amino acid at this position is located has an effect on binding to other molecules and interacts with a domain that is also involved in binding. The mutation of this position can disrupt these interactions and the function ofthe protein.	The amino acid is 100% conserved in this location. Thus, the mutation is generally harmful to the protein.
rs201122276	H1002R	The mutant residue is found in WD 2-3 repeated residues.	The domain in which the amino acid at this position is located has an effect on binding to other molecules and interacts with a domain that is also involved in binding. The mutation of this position can disrupt these interactions and the function ofthe protein.	The wild-type amino acid is very conserved; however, another amino acid type may be found at this location. Thus, the mutation is likely not harmful to the protein.
rs201911170	G951E	The mutant residue is found in WD 2-1 repeated residues. Mutation of glycine can damage the function of protein becauseglycine is the most flexible amino acid.	The domain in which the amino acid at this position is located has an effect on binding to other molecules and interacts with a domain that is also involved in binding. The mutation of this position can disrupt these interactions and the function ofthe protein.	The amino acid is 100% conserved in this location. Thus, the mutation is generally harmful to the protein.
rs202243082	P559T	The wild-type amino acid is proline, which is known to be very inelastic. The mutation of proline can disrupt the conformation of the protein in this position.	The amino acid at this position is situated on the surface of a domain that has unknown function.	The amino acid is 100% conserved in this location. Thus, the mutation is generally harmful to the protein.
rs371118366	C401R	The mutation occurs within the NB-ARC (UniProt) domain.	The domain in which the amino acid at this position is located has an effect on binding to other molecules and interacts with a domain that is also involved in binding. The mutation of this position can disrupt these interactions and the function of the protein.	The mutation can be harmful to the protein because the amino acid is located near a substantially conserved position.
rs371897242	R612C		The domain in which the amino acid at this position is located has an effect on binding to other molecules and interacts with a domain that is also involved in binding. The mutation of this position can disrupt these interactions and the function of the protein.	The mutant residue is located near a substantially conserved position but is probably not harmful for the protein due to some homolog sequences.
rs372400613	F544I		The amino acid is embedded in the core of a domain which can be disrupted due to mutation at this position.	The amino acid is 100% conserved in this location. Thus, the mutation is generally harmful to the protein.
rs373209321	D479G	The mutant amino acid is glycine, which is known to be very elastic. Therefore, the mutation can disrupt the rigidity of the protein at this position.	The amino acid is embedded in the core of a domain which can be disrupted due to mutation at this position.	The wild-type amino acid is frequently located at this position but another type of amino acid can be found here, too. However, the mutation is likely not harmful to the protein.
rs376175905	F547I		The amino acid at this position is situated on the surface of a domain which has unknown function.	The amino acid is 100% conserved in this location. Thus, the mutation is generally harmful to the protein.
rs147932236	S161F	The mutation occurs within the NB-ARC (UniProt) domain.	The domain in which the amino acid at this position is located has an effect on binding to other molecules and interacts with a domain that is also involved in binding. The mutation of this position can disrupt these interactions and the function of the protein.	The wild-type amino acid is very conserved; however, another amino acid type may be found at this location. Thus, the mutation is likely not harmful to the protein.
rs150457288	Y24C	The mutation occurs within the CARD (UniProt) domain.	The amino acid is embedded in the core of a domain which can be disrupted due to mutation at this position.	The wild-type amino acid is very conserved; however, another amino acid type may be found at this location. Thus, the mutation is likely not harmful to the protein.
rs200830441	H183R	The mutation occurs within the NB-ARC (UniProt) domain.	The domain in which the amino acid at this position is located has an effect on binding to other molecules and interacts with a domain that is also involved in binding. The mutation of this position can disrupt these interactions and the function of the protein.	The amino acid is 100% conserved in this location. Thus, the mutation is generally harmful to the protein.
rs201122276	H1002R	The mutant residue is found in WD 2-3 repeated residues.	The domain in which the amino acid at this position is located has an effect on binding to other molecules and interacts with a domain that is also involved in binding. The mutation of this position can disrupt these interactions and the function of the protein.	The wild-type amino acid is very conserved; however, another amino acid type may be found at this location. Thus, the mutation is likely not harmful to the protein.
rs201911170	G951E	The mutant residue is found in WD 2-1 repeated residues. Mutation of glycine can damage the function of protein because glycine is the most flexible amino acid.	The domain in which the amino acid at this position is located has an effect on binding to other molecules and interacts with a domain that is also involved in binding. The mutation of this position can disrupt these interactions and the function of the protein.	The amino acid is 100% conserved in this location. Thus, the mutation is generally harmful to the protein.
rs202243082	P559T	The wild-type amino acid is proline, which is known to be very inelastic. The mutation of proline can disrupt the conformation of the protein in this position.	The amino acid at this position is situated on the surface of a domain that has unknown function.	The amino acid is 100% conserved in this location. Thus, the mutation is generally harmful to the protein.
rs371118366	C401R	The mutation occurs within the NB-ARC (UniProt) domain.	The domain in which the amino acid at this position is located has an effect on binding to other molecules and interacts with a domain that is also involved in binding. The mutation of this position can disrupt these interactions and the function of the protein	The mutation can be harmful to the protein because the amino acid is located near a substantially conserved position.
rs371897242	R612C		The domain in which the aminoacid at this position is located has an effect on binding to other molecules and interacts with a domain that is also involved in binding. The mutation of this position can disrupt these interactions and the function of the protein.	The mutant residue is located near a substantially conserved position but is probably not harmful for the protein due to some homolog sequences.
rs372400613	F544I		The amino acid is embedded in the core of a domain which can be disrupted due to mutation at this position. The amino acid is 100% conserved in this location. Thus, the mutation is generally harmful to the protein.
rs373209321	D479G	The mutant amino acid is glycine, which is known to be very elastic. Therefore, the mutation can disrupt the rigidity of the protein at this position.	The amino acid is embedded in the core of a domain which can be disrupted due to mutation at this position.	The wild-type amino acid is frequently located at this position but another type of amino acid can be found here, too. However, the mutation is likely not harmful to the protein.
rs376175905	F547I		The amino acid at this position is situated on the surface of a domain which has unknown function.	The amino acid is 100% conserved in this location. Thus, the mutation is generally harmful to the protein.

## 4. Discussion

**Figure 4:**
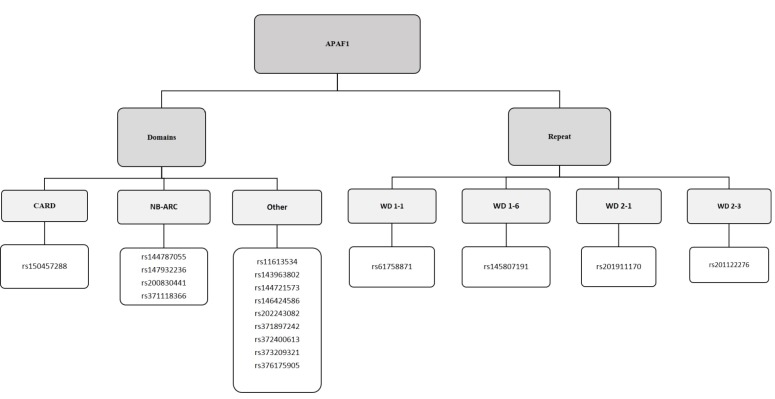
Schematic diagram of the APAF1 proteins and association with deleterious SNPs in APAF1 gene.

Project Hope software results have provided important information about the possible effects of missense SNPs in the *APAF1* gene. The *APAF1* rs144787055, rs147932236, rs200830441, and rs371118366 polymorphisms resulting in D170E, S161F, H183R, and C401R amino acid substitutions, respectively, were investigated within the NB-ARC domain (Table 4, Figure 4). These substituted amino acids have different physicochemical properties which could possibly disrupt the NB-ARC domain and eliminate its function. In addition, the wild-type aspartic acid residue at position 170 forms a hydrogen bond with glutamine at position 137 and serine at position 172, as well as a salt bridge with glycine at position 105. The SNP rs144787055 results in D170E substitution. The difference in size between the wild type and the substituted residue caused the new amino acid not to be in the correct position to form the original hydrogen bonds (Table 2). Furthermore, the wild-type serine at position 161 is apparently involved in a metal-ion contact in the APAF1 protein structure. An S161F substitution due to rs147932236 could possibly disrupt the metal-ion interactions, because phenylalanine is larger compared to the wild-type amino acid. In contrast to serine, the mutant amino acid could not form a hydrogen bond with aspartic acid at position 243 due to size and hydrophobicity (Table 2). The Project HOPE results showed that the wild-type amino acid serine at position 161 interacted with a ligand, DTP (like deoxyadenosine triphosphate). Mutation of serine to phenylalanine at this position might disturb the protein’s function, because ligand binding is important for the function of proteins. Cysteine at position 401 can form a hydrogen bond with threonine at position 408 (Table 4). The mutation of cysteine into an arginine (C401R) because of rs371118366 could affect hydrogen bond formation due to size and hydrophobicity (Table 2). The SNP rs150457288 resulted in cysteine to tryptophan substitution (Y24C) within the CARD domain (Table 4, Figure 4). The mutation of tyrosine into a cysteine at position 24 could possibly affect the structure and function of the CARD domain. The polymorphisms rs61758871, rs145807191, rs201911170, and rs201122276 were found to result in Y619S, S860L, G951E, and H1002R amino acid substitutions, respectively, within the tryptophan–aspartic acid (WD) 1-1, WD 1-6, WD 2-1, and WD 2-3 repeats, respectively (Table 4, Figure 4). These mutations might have a possible effect on the function of the WD repeats. In addition, the D443N mutation due to rs144721573 showed that the difference in charge between the wild-type and mutant amino acids could result in the disruption of ionic contacts, such as a salt bridge formed by the aspartic acid with lysine at position 348 and lysine at position 391 (Table 2).

According to the results of the GeneMANIA software tool, among the genes that *APAF1* interacted most with were *CASP9* (caspase 9), *CYCS* (cytochrome c, somatic), *BIK* (BCL2 interacting killer), *ANP32A* (acidic nuclear phosphoprotein 32 family member A), and *AVEN *(apoptosis and caspase activation inhibitor). Furthermore, GeneMANIA results showed that *APAF1*, *CASP9*, and *CYCS* genes have many important functional roles such as positive regulation of peptidase activity and the intrinsic apoptotic signaling pathway.

Thorough literature research shows that there have been a limited number of studies investigating the association of SNPs in the *APAF1 *gene with diseases. Zheng et al. (2016) examined the associations of SNPs in apoptotic pathway genes including *APAF1* with chronic myeloid leukemia; they reported no significant results for the impact of rs1439123 and rs2288713 on the *APAF1 *gene (Zheng et al., 2016). Choi et al. (2015) investigated the relationship between the pathology determinants of colorectal cancer and the frameshift mutations of the proapoptotic *APAF1*, *BAX*, and *FLASH* genes. They suggested that the genes might play a synergistic role in tumorigenesis (Choi et al., 2015). Enjuanes et al. (2008) reported that the rs17028658 polymorphism located in the 3’ region of the *APAF1* gene was significantly associated with a risk of chronic lymphocytic leukemia (Enjuanes et al., 2008).

## 5. Conclusions

The present study investigated the influence of functional SNPs associated with the *APAF1* gene via in silico methods because *APAF1* is related to many diseases such as cancer, neurodegenerative disorders, heart disease, autoimmunity, and immunodeficiencies. In a total of 18,084 SNPs in the *APAF1* gene, 772 SNPs were found to be missense. Furthermore, 18 SNPs were found to be deleterious or damaging by several software tools. We believe that our results will lay the foundation for future experimental and in silico studies.
